# Frequency of Post-operative Surgical Site Infections in a Tertiary Care Hospital in Abbottabad, Pakistan

**DOI:** 10.7759/cureus.4243

**Published:** 2019-03-12

**Authors:** Farhan Sattar, Zeeshan Sattar, Mohsin Zaman, Shahzad Akbar

**Affiliations:** 1 Internal Medicine, Ayub Teaching Hospital, Abbottabad, PAK; 2 Internal Medicine, Khyber Teaching Hospital, Peshawar, PAK; 3 General Surgery, Khyber Teaching Hospital, Peshawar, PAK; 4 Geriatrics, Hull and East Yorkshire Hospitals National Health Service Trust, Hull, GBR

**Keywords:** surgical site infection, incidence

## Abstract

Background

Any infection occurring in a wound after a surgical procedure is called a post-operative surgical wound infection. Surgical wound infection is a type of nosocomial infection that is acquired in hospitals when a person is admitted for reasons other than the infection. Surgical site infections (SSIs) are the second most common nosocomial (hospital-acquired) infections after urinary tract infections. SSIs contribute to a significant rate of morbidity and mortality in patients and has become a major public health problem worldwide. The increase in antibiotic resistance associated with SSIs has also become a therapeutic challenge for physicians worldwide.

Methods

This cross-sectional study was carried out among the patients admitted in various surgical wards of the Ayub Teaching Hospital. A total of 95 patients were enrolled for the study using a non-probability convenient sampling technique. Data were collected using a structured questionnaire by students carrying out the research for a time period of one month. SSIs were assessed by:

1. Pus or purulent discharge from the wound along with pain,

2. Any two cardinal signs of inflammation, and

3. Diagnosis of SSI by the surgeon.

Results

The mean age of the patients was 35.73 ± 19.73 years. SSI rate was found to be 33.68% with 32 patients developing SSIs out of 95 patients. The rate of SSIs was greater in older patients with four (44.4%) out of nine patients above 60 years developing SSIs. Patients belonging to urban areas had a higher incidence rate (52.77%) of SSIs as compared to rural areas (32.20%). The rate of SSIs in patients operated with an elective surgical plan was greater (37.93%) as compared to patients operated with an emergency surgical plan (27.77%). Patients who were obese were more prone to SSIs with seven (36.8%) patients developing SSIs out of 19. Surgeries performed by trainee medical officers had a greater rate of SSIs with 24 (35.3%) patients developing SSIs out of 68 patients. Three (66.66%) out of four patients with diabetes and 18 (40.9%) out of 44 patients with anemia developed SSIs.

Conclusion

This study concluded that the incidence rate of SSIs was high in patients admitted in Ayub Teaching Hospital. Major risk factors identified were co-morbidities, old age, obesity, duration of surgery, major surgeries, and anemia. Steps should be taken to decrease SSIs in these high-risk groups.

## Introduction

Infection of a wound after a surgical operation is called post-operative surgical wound infection. The rates of these infections vary from hospital to hospital, and the site of the infection may be limited to the suture line or may extend into the operative site. Surgical wound infection is a type of nosocomial infection [1]. Nosocomial infections are those infections that are acquired in hospitals or other healthcare facilities. For a person to have acquired a nosocomial infection, he or she must be admitted to a hospital or healthcare facility for reasons other than the infection and no sign of active or incubating infection should be shown by the patient [2].

Nosocomial infections can be urinary tract infections, respiratory infections, or surgical wound infections [3]. Surgical wound infection is the most common nosocomial infection after urinary tract infection [4]. These infections account for 20% to 39% of all the infections acquired in hospitals [5]. Postoperative wound infection can occur from the first day onwards to many years after an operation but commonly occurs between the fifth and tenth days after surgery [6].

Wound infection is the commonest and most troublesome disorder delaying wound healing. When there is a breakdown of local and systemic host defenses followed by an invasion of organisms through tissues then the wound is said to be infected. When a wound discharges pus or needs a secondary procedure to be sure of adequate drainage, then the infection is regarded as a major wound infection. However, in minor wound infections, there is a discharge of serous fluid or pus but without any systemic signs or excessive discomfort [7].

The term “surgical wound infection” was replaced by “surgical-site infection” by the Surgical Wound Infection Task Force in 1992 to include infections of organs or spaces deep in the skin and soft tissues [8].

Surgical-site infections (SSIs) are classified by The Centre for Disease Control (CDC), USA into (a) superficial incisional SSI, (b) deep incisional SSI, and (c) organ/space SSI [9].

Various bacteriological studies reveal that both gram-positive and gram-negative bacteria play a role in the infection of surgical wounds [4]. The most common of these bacteria is *Staphylococcus aureus* (31.58%) followed by *Klebsiella pneumonia* (26.31%), *Pseudomonas aeruginosa* (15.79%), *Escherichia coli* (10.53%), *Acinetobacter* (10.53%) and *Proteus mirabilis* (5.26%) [10].

These bacteria pose a major problem for surgeons because most of them are multi-drug-resistant bacteria [10].

Postoperative SSIs can be quite lethal, remaining as a less frequent cause of mortality but a major source of morbidity in surgical patients. They account for approximately one-quarter of the estimated two million nosocomial infections occurring yearly [11]. Furthermore, SSIs cause an increase in treatment cost, bed occupancy in a ward and prolong the hospital stay of the patient. In developing countries, due to limited resources, even basic life-saving procedures like appendectomies and cesarean sections are associated with high infection rates of wounds and mortality [10]. In orthopedic surgeries like joint replacement and internal fixation of the spine, complications due to postoperative sepsis can lead to calamitous consequences [12].

Various factors affecting the infection rate include skin preparation, wound contamination, the length of pre-operative hospital stay, drainage of wounds, the age of the patient, duration of surgery, and skill and technique of the surgeon. There is higher infection rate involving senior surgeons which can be attributed to the fact that they perform more difficult and lengthy surgeries, while the low rate of infections in surgeries performed by medical officers can be attributed to the fact that they perform simpler and uncomplicated surgeries [12].

In many developing countries including Pakistan, a properly organized surveillance system to describe routine SSI rates does not exist. Infection of surgical sites is a burden, almost every hospital in Pakistan has to deal with. Success in surgery depends on prevention and proper management of a wound. In order to adapt the policies which decrease the incidence of SSIs, the most important requirement is to collect data, perform wound surveillance and surgical inspection. Unfortunately, this aspect of surgery is the least discussed topic in local literature and we have to refer to the Western literature for the incidence of SSIs. Being the only tertiary care hospital of Abbottabad, Ayub Teaching Hospital (ATH) has to deal with a greater degree of patient burden which subsequently increases the various risks associated with SSIs. A study conducted to find the frequency of various post-operative SSIs at ATH can prove to be useful.

## Materials and methods

A cross-sectional study was performed in the surgery and allied, gynecology wards B and C and orthopedics ward of the Ayub Teaching Hospital, Abbottabad from April to May 2016. The sample size was 95 sampled through the non-probability convenient sampling technique.

All the patients admitted in the surgery and allied, gynecology ward (B and C), and orthopedics ward, who had undergone surgery (major or minor) and their post-operative stay was longer than three days, were included. Those patients were also included who were admitted in the wards due to development of SSIs after discharge and their treatment was not started. Patients of all age and both genders were included.

Those patients who became serious after surgery and/or who developed SSIs after discharge and admitted for treatment and their treatment was started were excluded.

A structured questionnaire was developed including several variables of interest. Students visited the surgical, gynecology and orthopedic wards and collected information from two chambers of males and two chambers of females of the same ward. The questionnaires were filled by students. Informed consent was taken from all the patients and confidentiality of data was ensured.

Surgical wounds were observed by students. SSIs were assessed and wounds were considered infected when pus/purulent discharge and pain along with any other sign of inflammation in the wound was found or when a diagnosis was made by the surgeon. During the assessment of the wound, an aseptic technique was used to prevent the contamination of the wounds.

The data were entered and analyzed using computer software SPSS version 16.0. Quantitative variables such as age, pre-op hospital stay, and duration of surgery were described as mean ± SD and categorical variables like gender, anemic/non-anemic state and category of surgeon were described as frequency and proportion. Data were presented in tables and figures.

## Results

The total study population was 95 patients who underwent surgery in the surgical wards (A, B, and C), orthopedics ward, and gynecology wards (B and C) during a period of one month at ATH. Out of the 95 patients, 41 (43.2%) were male and 54 (56.8%) were female. Male to female ratio was 0.75. The mean age of the patients was 35.73 ± 19.73 years with a minimum age of one year and maximum age of 100 years. Out of the 95 patients, 58 (61.1%) were married, 29 (30.5%) were single, seven (7.4%) were widowed, and one (1.1%) was divorced. Patients who belonged to rural areas were 58 (61.1%) and those who belonged to urban areas were 37 (38.9%). Fifty-eight (61.19%) patients were illiterate, while 37 (38.81%) were literate. Eighty-four (88.4%) patients were in the lower class, 10 (10.5%) patients were in the middle class, while only one (1.1%) patient belonged to the upper class. The number of elective procedures was 58 (61.1%), while 37 (38.9%) cases were performed as emergency procedures.

**Figure 1:**
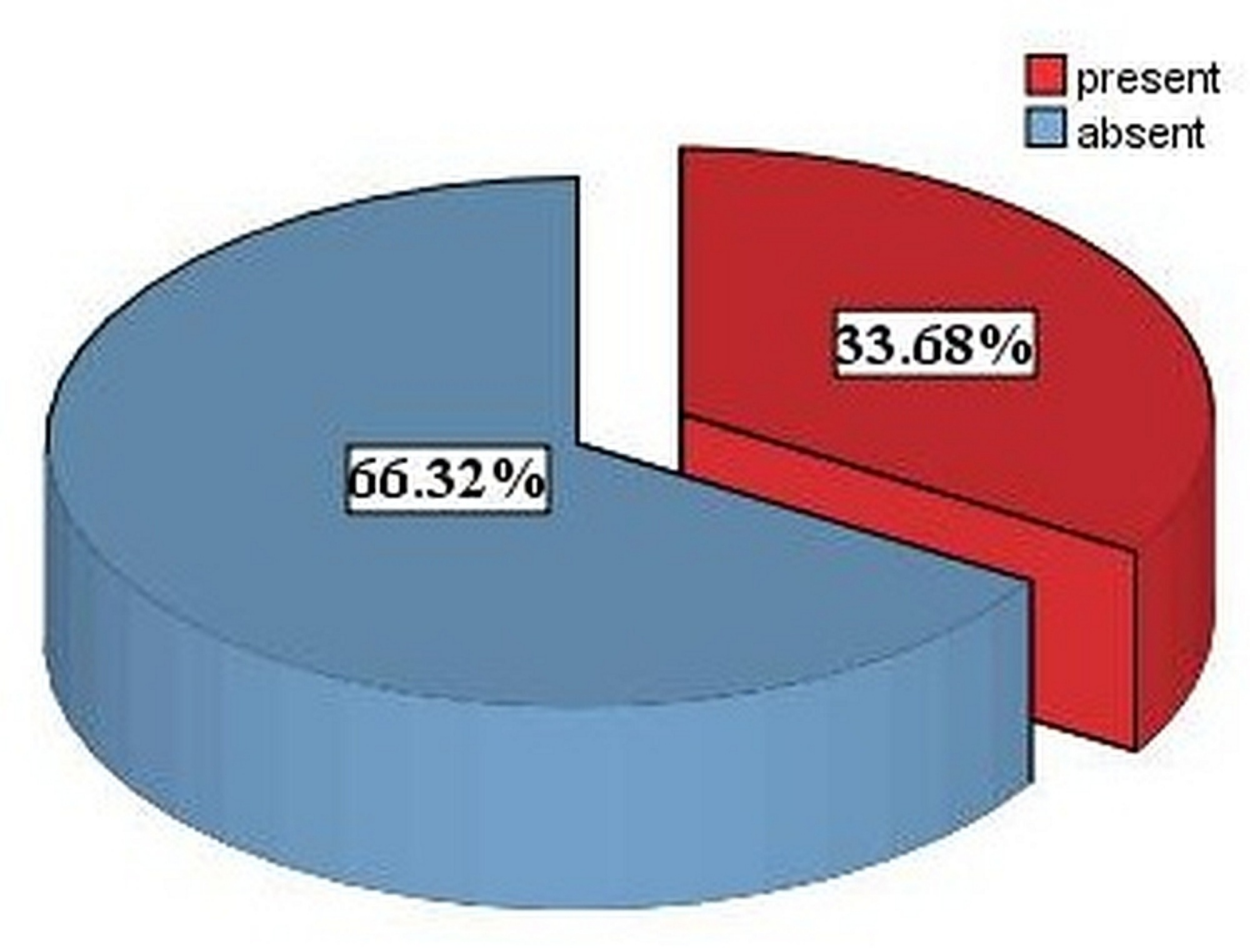
Frequency of SSIs in patients SSIs, surgical site infections

Figure 1 shows that out of 95 patients 32 (33.68%) developed SSI. Sixty-three (66.31%) patients did not develop SSIs.

**Figure 2:**
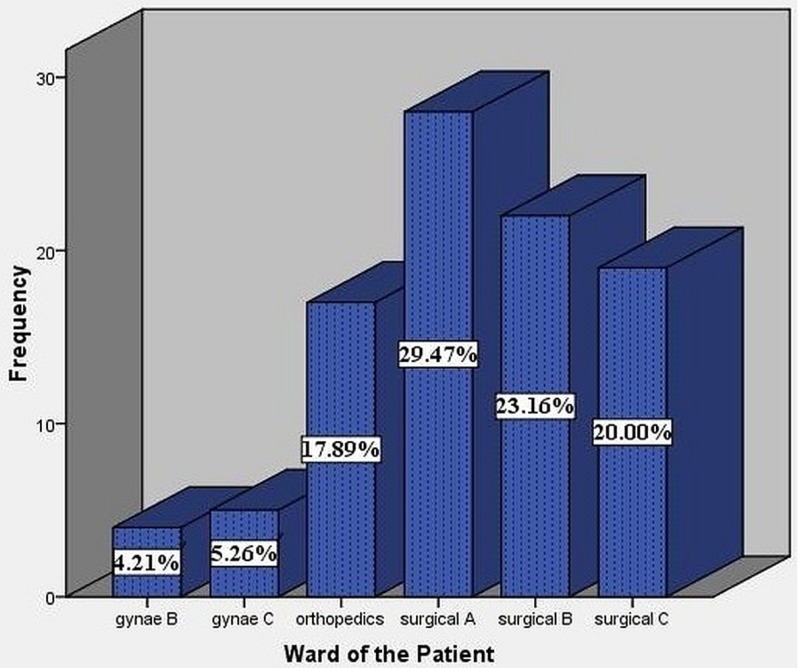
Distribution of the study population in various wards of ATH ATH, Ayub teaching hospital

The total study population was 95. Data were collected from different wards at the Ayub Teaching Hospital. The bulk of the data was collected from surgical ward A which was 28 (29.47 %), then from surgical B which was 22 (23.16%), surgical C 19 (20%), orthopedics 17 (17.89%), gynecology C five (5.26%), and gynecology B four (4.21%). In our study, eight (28.57%) out of 28 patients developed SSIs in surgical ward A of ATH, nine (40.90%) out of 22 in surgical ward B, six (31.57%) out of 19 in surgical ward C, seven (41.17%) out of 17 in orthopedics ward, two out of five (40%) from gynecology ward C, and out of the four patients, there were no cases SSIs in gynecology ward B. These data are summarized in Figure 2.

The mean age of the patients was 35.73 ± 19.73. The frequency of patients was maximum (49) from the age group 15-40 years and minimum (nine) from patients whose age was above 60 years. Table 1 shows the age distribution frequency of the patients who were interviewed. From patients whose age was less than 15 years, one (9.09%) patient developed SSI; from age group 15-40 years, 18 (36.73%) patients developed SSIs; from 41-60 years, nine (34.61%) patients developed SSIs; and from patients whose age was above 60 years, four (44.44%) patients developed SSIs.

**Table 1:** Frequency of SSIs in different age groups SSIs, surgical site infections

Age Groups (In Years)	Frequency	SSIs
Less than 15	11	1 (9.09%)
15-40	49	18 (36.73%)
41-60	26	9 (34.61%)
Above 60	9	4 (44.44%)
Total	95	32 (33.68%)

**Figure 3:**
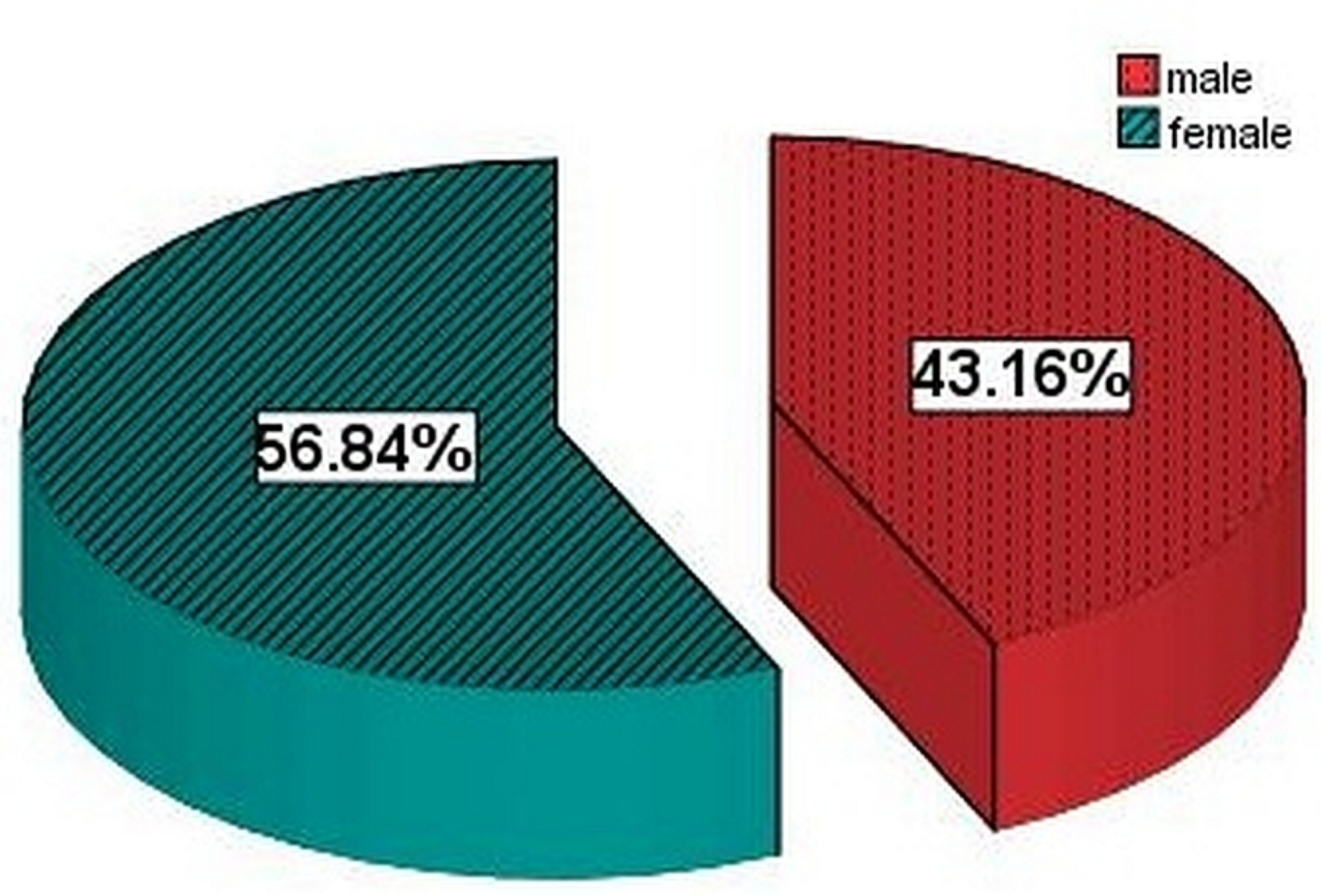
Gender distribution of the study population

In our study population, 41 patients were male (43.16%) and 54 were female (56.84%). Out of 41 males, 14 (34.14%) developed SSIs and 18 (33.33%) females developed SSI out of 54 (Figure 3).

**Figure 4:**
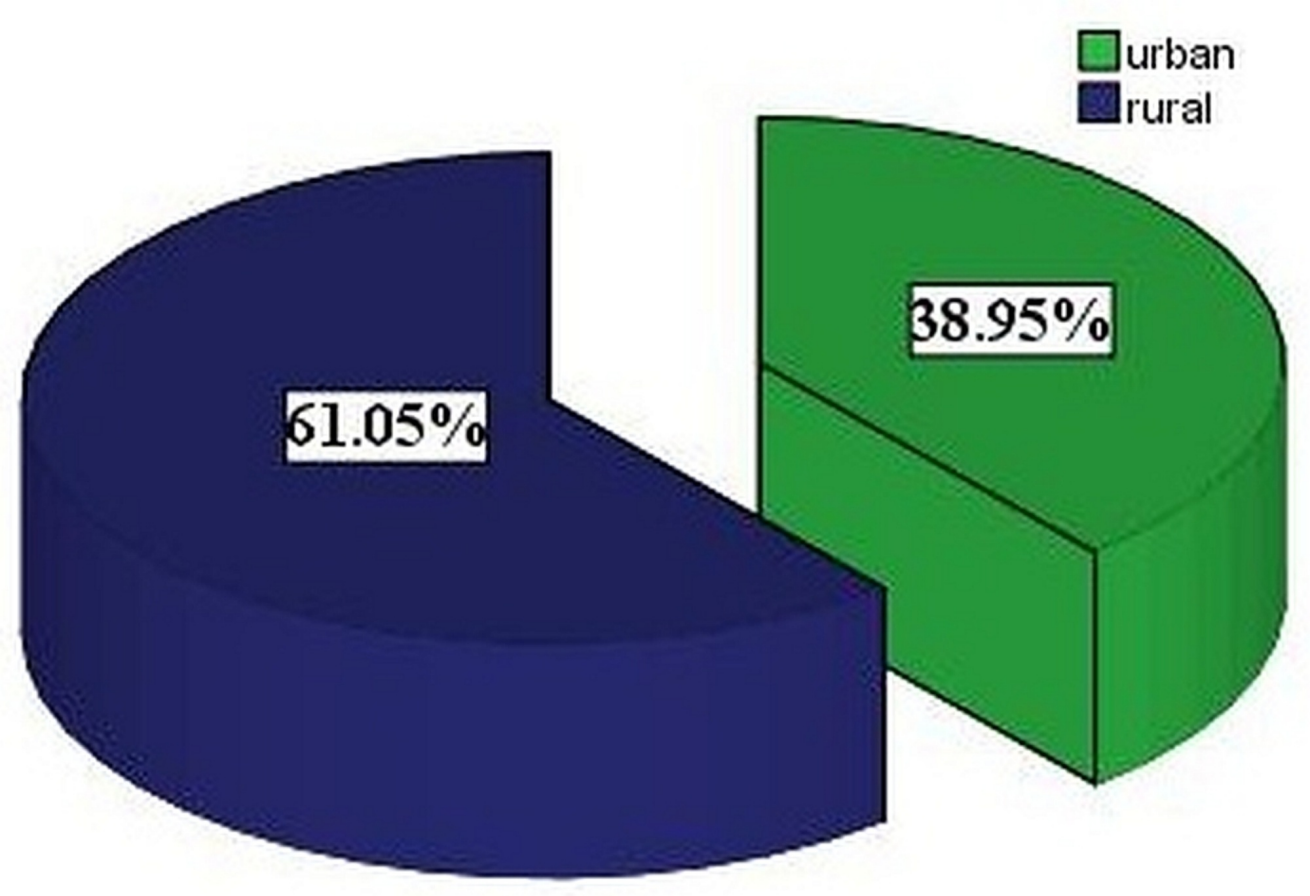
Area of residence

Out of the 95 patients, 58 (61.05%) belonged to rural areas and 37 (38.95%) to urban areas. From rural areas, 19 (32.20%) developed SSIs out of 59 and from urban areas 19 (52.77%) developed SSIs out of 36 (Figure 4).

**Figure 5:**
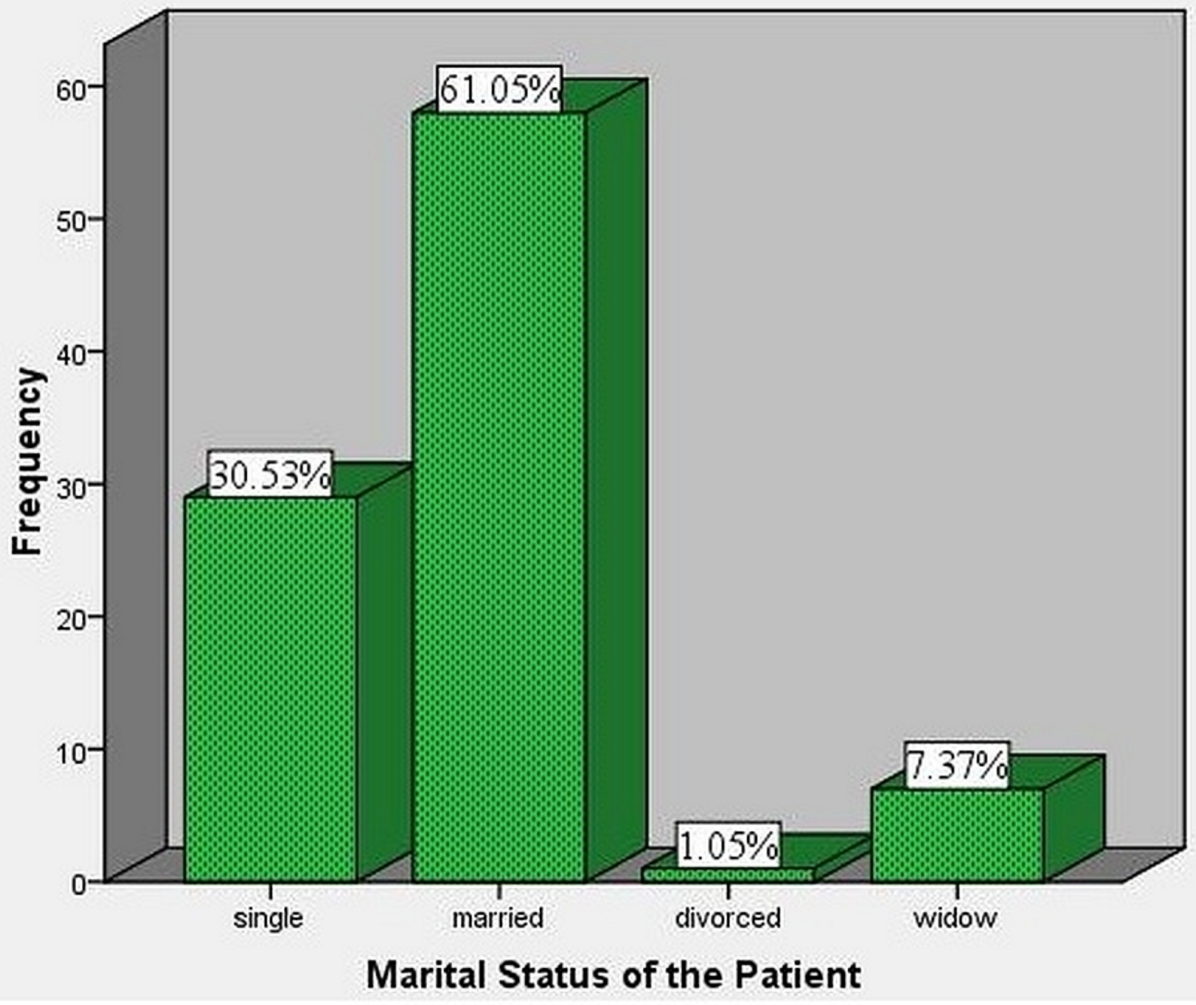
Marital status of the patients

Out of the study population of 95 patients, 58 (61.05%) patients were married, 29 (30.53%) were single, seven (7.37%) were widowed and one (1.05%) was divorced (Figure 5). Out of 58 married patients, 25 (43.10%) developed SSIs and in patients who were single, seven (24.13%) out of 29 developed SSIs. There were no SSIs in the seven widowed patients and one divorced patient.

Out of the 95 patients, 21 (22.1%) were unemployed, two (2.1%) were employed by the government, 10 (10.5%) had a private employment, four (4.2%) had their own business, 43 (45.3%) females were housewives, 12 (12.6%) patients were students and three (3.2%) were retired. Five (23.80%) out of 21 developed SSIs from the unemployed group. Sixteen (37.20%) out of 43 from housewives, six (60%) out of 10 from private employees, two (16.66%) out of 12 from students, two (66.66%) out of three retired, and one (50%) out of two developed SSIs from government employee group, respectively. These data have been summarized in Table 2.

**Table 2:** Occupation of the patients SSIs, surgical site infections

Occupation	Frequency (%)	SSIs
Unemployed	21(22.1%)	5 (23.80%)
Government Employee	2(2.1%)	1 (50%)
Private Employee	10(10.5%)	6 (60%)
Business	4(4.2%)	0
Housewife	43(45.3%)	16 (37.20%)
Student	12(12.6%)	2 (16.66%)
Retired	3(3.2%)	2 (66.66%)

**Figure 6:**
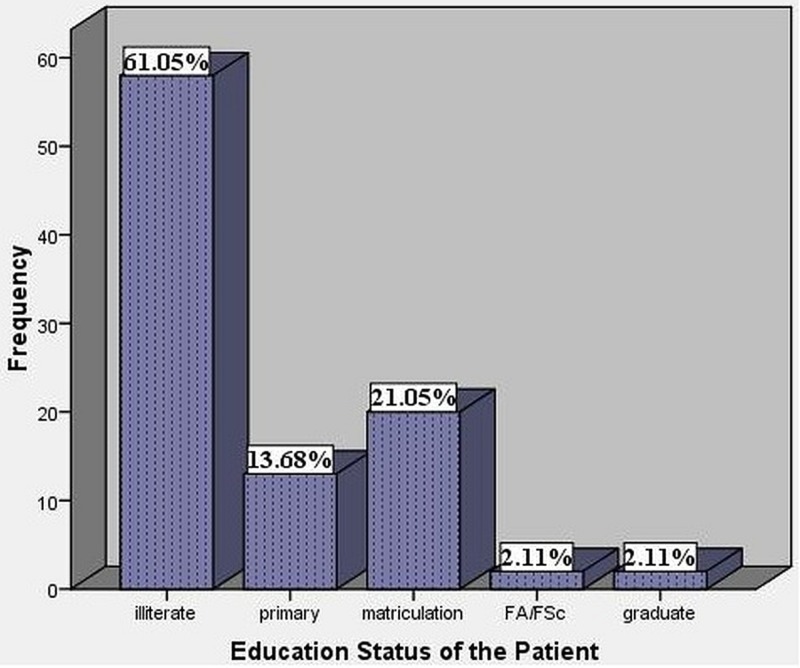
Education status of the patients

Most of the patients in our study were Illiterate consisting of 58 (61.05%) patients. Among the educated 13 (13.68%) were primary, 20 (21.05%) were matriculation, two (2.11%) were FA/FSc. and two (2.11%) were graduates (Figure 6). Among the illiterate patients, 19 (32.75%) developed SSIs. Five (38.46%) from primary, seven (35%) from matriculation, and one (50%) each from FA/FSc and graduate level developed SSIs, respectively. So, 13 (35.13%) literate people developed SSIs.

**Figure 7:**
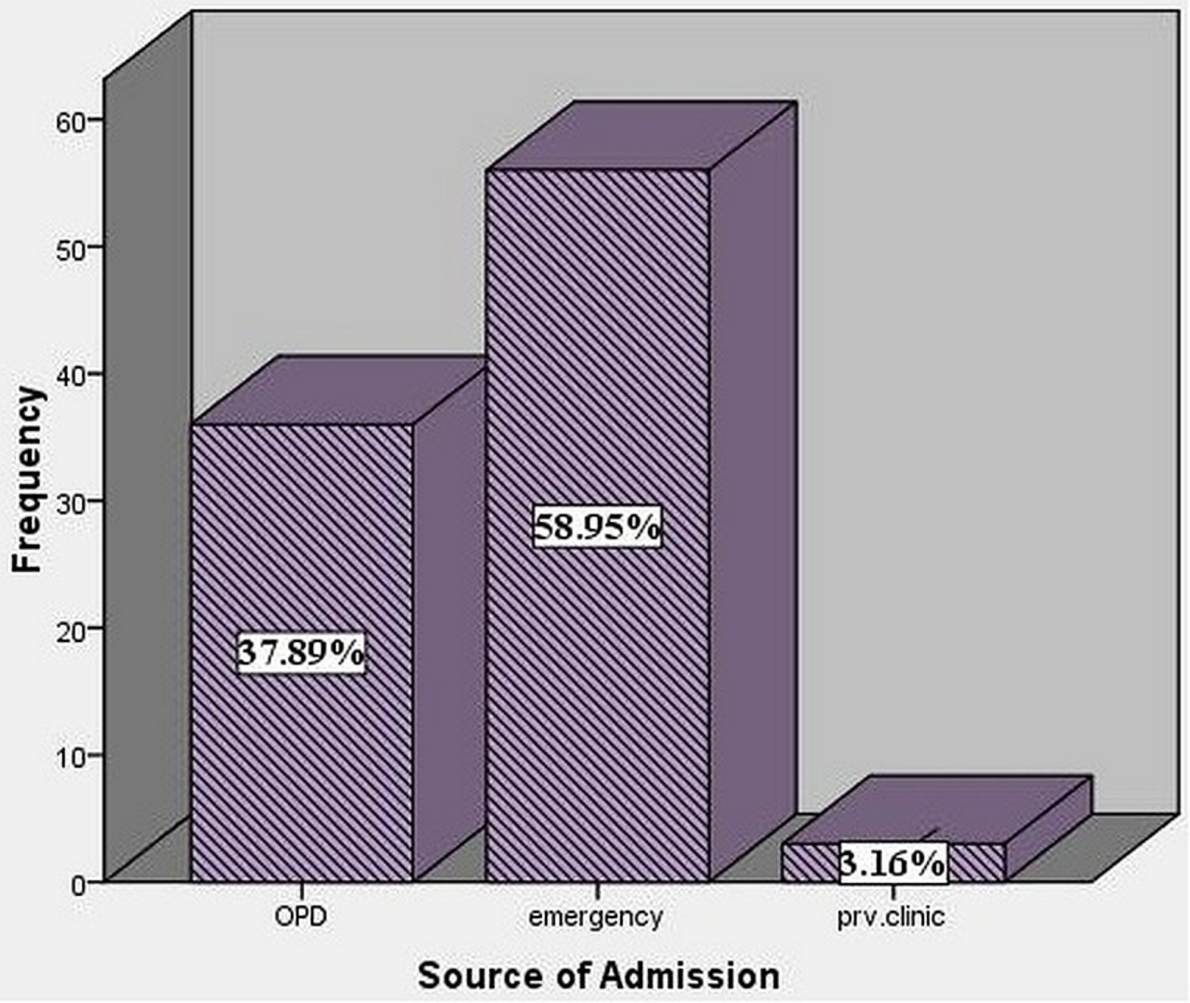
Source of admission of the patients

Out of the total study population, 56 (58.95%) patients were checked through emergency, 36 (37.89%) through O.P.D and three (3.16 %) were referred from private clinics (Figure 7). Ten (28.57%) patients from OPD developed SSIs, 19 (35.18%) patients developed SSIs from emergency and three (100%) out of three patients from private clinics developed SSIs.

Out of the 95 patients, 58 (61.1%) were planned for elective surgery while 36 (37.9%) were operated emergently. From patients with an elective surgical plan, 22 (37.93%) patients developed SSIs, while 10 (27.77%) patients developed SSIs from patients with an emergent surgical plan (Table 3). The association between the plan of surgery and SSIs was statistically insignificant (*p* = 0.31).

**Table 3:** Plan of surgery SSIs, surgical site infections

Plan	Frequency (%)	SSIs (%)
Elective	58(61.1%)	22(37.9%)
Emergency	36(37.9%)	10(27.7%)
Total	95(100%)	32(33.6%)

As given in Table 4 below, 25 patients (26.3%) presented with gastrointestinal problems. Seventeen patients (17.9%) presented with orthopedic problems, 13 (13.7%) with hepatobiliary, nine (9.5%) with gynecological, six (6.3%) with genitourinary, while 25 patients (26.3%) were categorized under "other" category like firearm injuries, etc. Eleven (44%) patients developed SSIs with gastrointestinal problem. Seven (41.17%) patients developed SSIs with orthopedic problems. Four (30.76%) patients with hepatobiliary, one (11.11%) patient with gynecological, one (16.66%) patient with genitourinary, and eight (32%) patients with other surgical problems developed SSIs, respectively.

**Table 4:** Surgical problems of the patients

Surgical problem	Frequency(%)	SSIs
Orthopedics	17(17.9%)	7 (41.17%)
Gynecological	9(9.5%)	1 (11.11%)
Gastrointestinal	25(26.3%)	11 (44%)
Hepatobiliary	13(13.7%)	4 (30.76%)
Genitourinary	6(6.3%)	1 (16.66%)
Others	25(26.3%)	8 (32%)
Total	95(100%)	32 (33.68%)

Table 5 shows the duration of the current illness of patients in days. It also shows the frequency of SSIs in different groups according to the duration.

**Table 5:** Total duration of current illness SSIs, surgical site infections

Duration	Frequency (%)	SSIs (%)
Below 30 days	80(84.2%)	25(31.2%)
30 to 60 days	3(3.2%)	3(100%)
Above 60 days	12(2.6%)	4(33.3%)
Total	95(100%)	32(33.6%)

As can be seen in Table 6, a bulk of the study population was in the lower economic class i.e. 84 (88.4%). Ten (10.5%) patients were in the middle economic class and only one patient (1.1%) was in the upper economic class. From the lower class, 28 (33.33%) patients developed SSIs, while four (40%) patients developed SSIs from the middle class. No patient from the upper class developed SSIs. The association between socioeconomic status and SSIs was statistically insignificant (*p* = 0.708).

**Table 6:** Relationship of SSIs with socioeconomic status of the patients SSIs, surgical site infections

Socio-economic Status	Surgical site infection		Total
	Present	Absent	
Lower Class	28 (33.3%)	56 (66.7%)	84 (88.4%)
Middle Class	4 (40%)	6 (60%)	10 (10.5%)
Upper Class	0 (0%)	1 (100%)	1 (1.1%)
Total	32 (33.7%)	62 (66.7%)	95 (100%)

Table 7 shows that most of the patients were not obese. Out of the 95 patients, 76 (80%) did not have obesity while 19 (20%) could be classified as obese. Seven (36.84%) patients who were obese developed SSIs while 25 (32.89%) patients who were not obese developed SSIs. The association between obesity and SSIs was statistically insignificant (*p* = 0.745).

**Table 7:** Relationship of SSIs with obesity in the patients SSIs, surgical site infections

Obesity	Surgical Site Infection		Total
	Present	Absent	
Present	7 (36.8%)	12 (63.2%)	19 (20%)
Absent	25 (26.3%)	51 (53.7%)	76 (80%)
Total	32 (33.7%)	63 (66.3%)	95 (100%)

**Figure 8:**
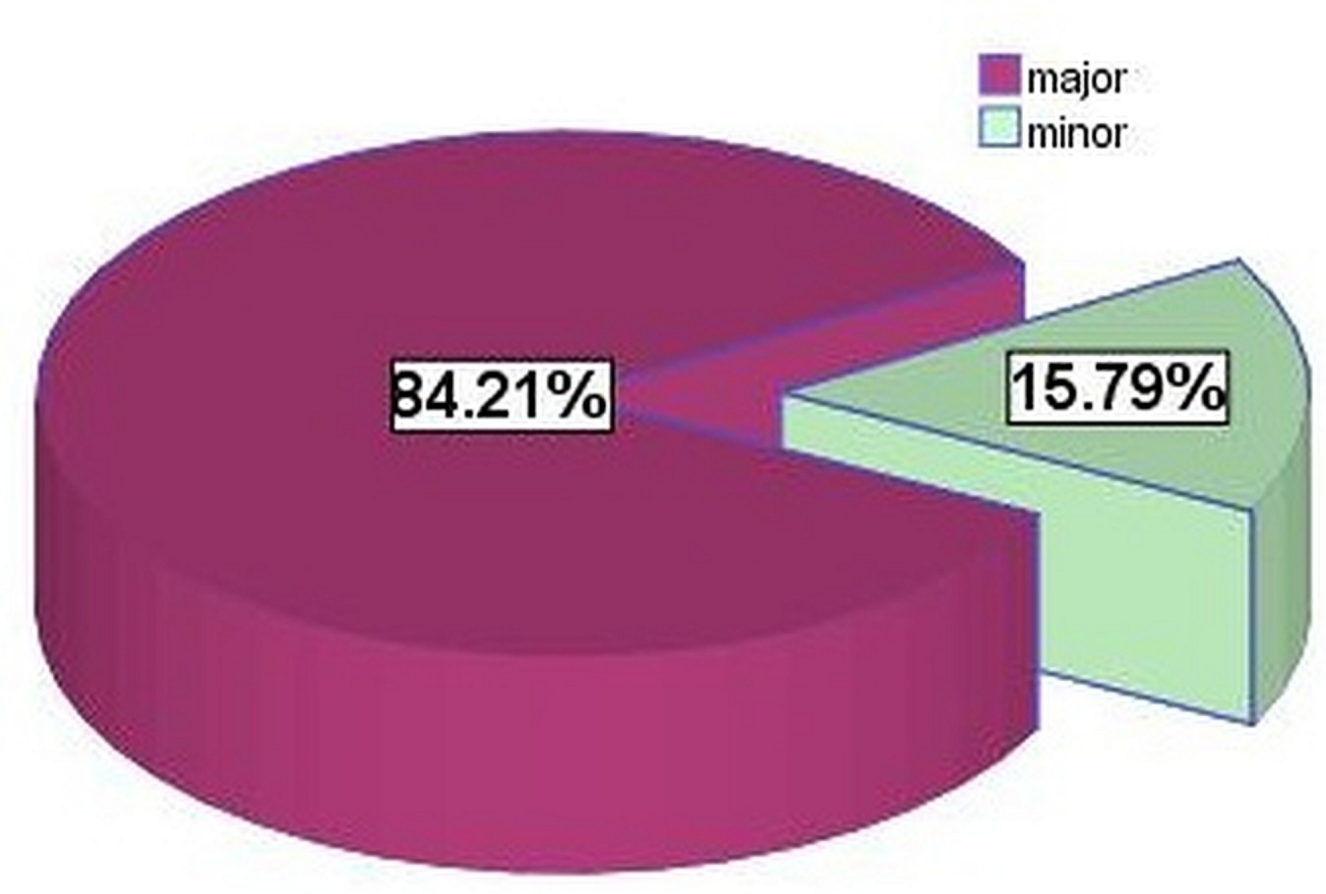
Surgical procedure done

Out of the 95 patients that we interviewed, 80 (84.2 %) had undergone a major procedure while 15 (15.8 %) had undergone a minor procedure (Figure 8). Twenty-eight (35%) patients who had undergone major surgeries developed SSIs, while four (26.66%) patients developed SSIs with minor surgeries.

Table 8 shows the frequency of SSIs in patients with different durations of surgery. The mean of the duration of surgery was 124.05 ± 74.26 minutes with a minimum of 15 minutes and a maximum of 360 minutes. The association between duration of surgery of patients and SSIs was statistically insignificant (*p* = 0.76).

**Table 8:** Duration of surgery

Duration(min)	Frequency	SSIs
Below 60	15 (15.78%)	4 (26.66%)
60-90	24 (25.26%)	8 (33.33%)
Above 90	56 (58.94%)	20 (35.71%)
Total	95 (100%)	32 (33.68%)

Most of the cases, i.e., 68 (71.6%) were managed by training medical officers (TMOs), 19 were managed by registrars (20%), six (6.3%) were managed by assistant professors, while two (2.1%) cases were managed by associate professors. Twenty-four (35.29%) patients operated by the TMOs developed SSIs, six (31.57%) patients operated by registrars developed SSIs, one (16.66%) patient operated by assistant professor developed SSI, and one (50%) patient by associate professor developed SSI. The association between SSIs and category of the surgeon was statistically insignificant (*p *= 0.76). These data have been summarized in Table 9.

**Table 9:** Category of surgeon

Category of Surgeon	Surgical Site Infection		TOTAL
	Present	Absent	
Registrar	6 (31.6%)	13 (66.4%)	19
TMO	24 (35.3%)	44 (64.7%)	68
Assistant Professor	1 (16.7%)	5 (83.3%)	6
Associate Professor	1 (50%)	1 (50%)	2
Professor	0 (0%)	0 (0%)	0
Total	32 (33.7%)	63 (66.3%)	95

Table 10 shows the number of SSIs in patients with different durations of surgery. The mean of their pre-op hospital stay is 76.13 hours with a minimum of one hour and a maximum of 360 hours. The Standard Deviation is 91.222. The association between the pre-op hospital stay and SSIs was statistically insignificant (p=0.93).

**Table 10:** Pre-op hospital stay SSIs, surgical site infections

Pre-op Hospital stay (in hours)	Frequency	SSIs
Below 36	51	15 (29.41%)
36-72	10	5 (50%)
73-146	18	6 (33.33%)
Above 146	16	6 (37.5%)
Total	95	32 (33.68%)

As can be seen from Table 11, most of the patients i.e. 77 (81.1%) did not suffer from any associated morbidity. Three (3.2%) patients had diabetes, three (3.2%) had renal failure and 12 (12.6%) patients had other morbidities like hepatitis, hypertension, etc. The number of patients who developed SSIs and had no associated morbidity was 24 (31.16%). In patients with diabetes, two (66.66%) patients developed SSIs and in patients with other morbidities, six (50%) developed SSIs. The percentage of patients with associated morbidities who developed SSIs was 44.44%. The association between associated-morbidity and incidence of SSIs was statistically significant (*p* = 0.04).

**Table 11:** Associated morbidity in patients SSIs, surgical site infections

Morbidity	Frequency	SSIs
Nil	77 (81.1%)	24 (31.16%)
Diabetes	3 (3.2%)	2 (66.66%)
Renal Failure	3 (3.2%)	0
Other	12 (12.6%)	6 (50%)
Total	95	32 (33.68%)

According to Table 12, 44 (46.3%) patients were anemic while 51 (53.7%) patients were non-anemic. Eighteen (40.90%) patients who were anemic developed SSIs and 14 (27.45%) patients who were non-anemic developed SSIs. The association between anemia in patients and SSIs was statistically insignificant (*p* = 0.16).

**Table 12:** Anemia in patients

ANEMIA IN PATIENTS	SURGICAL SITE INFECTION		TOTAL
	PRESENT	ABSENT	
PRESENT	18 (40.9%)	26 (59.1%)	44 (46.3%)
ABSENT	14 (27.5%)	37 (72.5%)	51 (53.7%)
TOTAL	32 (33.7%)	63 (66.7%)	95 (100%)

## Discussion

Infection of wounds after surgical operations is a real risk associated with any surgical procedure and represents a significant burden in terms of patient morbidity and mortality. We conducted a study at the Ayub teaching hospital to find the frequency of SSIs, its association with the type and site of surgery, and risk factors associated with SSIs. Our study showed the proportion of SSIs to be 33.68%, i.e., 32 patients out of 95 patients. A study conducted in public hospitals of Yemen showed similar results in which 300 patients were interviewed and 34% suffered from SSIs [13].

Our study showed that SSIs were greater in patients whose age was above 60 years with a percentage of 44.44% and lower in patients whose age was less than 15 years with a percentage of 9.09% showing that as the age increases the risk of SSI in the patient also increases. A study conducted at Andhra Pradesh, India showed similar results [14]. Increasing age is associated with a greater likelihood of certain chronic conditions and delayed healing which is most probably the cause of the increased incidence in higher age groups.

Our study showed a slightly greater percentage (34.14%) of SSIs in males than females (33.33%). No statistically significant relation was found between the development of SSIs and marital status, educational status, socioeconomic class, and occupation; similar to a study conducted in Africa [15].

Obesity is another important patient-related risk factor. Our study concluded that the incidence of SSIs in patients with obesity was higher than non-obese patients. Morbid obesity has been correlated with prolonged wound healing which is a known risk factor for deep SSIs [16].

Most patients presented with gastrointestinal and orthopedic problems. SSI rates were maximum in gastrointestinal surgeries (44%) and minimal in genitourinary surgeries (16.66%). A study conducted at the Khyber Teaching Hospital, Peshawar also showed SSI rates to be greater in gastrointestinal surgeries and minimal in genitourinary surgeries [17]. The surgical procedure done on these patients was classified into major and minor. SSI rates were higher in major surgeries as compared to minor surgeries. The result is similar to a study conducted in a tertiary care hospital in Africa showing that SSI risk in major surgeries is higher [15].

Inspecting the duration of surgeries and their relation with SSIs showed that the percentage of SSI was greater in surgeries that took more than 90 minutes. This observation was probably due to the fact that complex surgeries take more time which increases the risk for SSIs accordingly. A study performed at the Peoples Medical College and Hospital, Nawab Shah also showed that SSIs were greater in surgeries whose duration was greater than 80 minutes [18].

Regarding the plan of surgery, our study showed that patients who had an elective procedure had a higher rate (37.93%) than those who had an emergency procedure (27.77%). Another study showed SSI rates to be higher in emergency procedures [15]. The contradictory results are most probably due to the fact that in our study most of the surgical procedures were performed by TMOs who have less experience than professors. The rate of SSIs was higher in surgeries performed by TMOs (31.57%), and the incidence decreased with more experienced surgeons.

Patients with diabetes as a co-morbidity had a greater percentage of SSIs (66.66%) as compared to other co-morbidities such as renal failure and hypertension. A study conducted at a teaching hospital in Saudi Arabia also showed diabetes to be an important risk factor in the incidence of SSIs, in which 20 out of 80 patients with diabetes developed an infection after surgery [19]. Similarly, anemia was also found to be a risk factor. Out of the 51 patients who were anemic, 18 (40.90%) developed an infection after surgery. A study conducted at Nawab Shah also showed anemia to be a risk factor in the incidence of SSIs [18].

Our study showed that patients with a pre-operative stay of 36-72 hours showed the maximum percentage of SSIs (50%) followed by patients with a pre-operative hospital stay above 146 hours. A study conducted at the United States showed pre-operative hospital stay as a significantly associated risk factor. According to the study, as the pre-operative hospital stay increases, the incidence of SSIs increases accordingly [20].

## Conclusions

Our study concluded that the percentage of SSIs in the Ayub Teaching Hospital was 33.68%, i.e., 32 out of 95 patients developed SSIs. Associated morbidity was found to be the major risk factor. The association between associated morbidity and incidence of SSIs was found to be statistically significant (*p* = 0.04). The rates of SSIs were also found to be greater in older patients, patients with an elective surgical plan, obese patients, patients with long duration of surgeries, patients who had undergone major surgical procedures, patients operated by TMOs, and patients with anemia. These can be considered as suspected risk factors. Steps should be taken to decrease SSIs in these high-risk groups. Some recommendations to decrease SSIs are given below.

1. Patients and their caretakers should be given proper information and advice on how to care for their wound after discharge, how to recognize an SSI and whom to contact if they are concerned.

2. Proper antibiotic prophylaxis should be given when necessary.

3. Surgeons and other operating theater personnel should follow proper aseptic techniques to prevent contamination of the wound.

4. Changing wound dressing and its care should be done by properly trained nurses.

5. Patients should be advised to have a bath using soap either the day before or on the day of surgery.
